# Mesenchymal Stem Cells Coated by the Extracellular Matrix Promote Wound Healing in Diabetic Rats

**DOI:** 10.1155/2019/9564869

**Published:** 2019-01-28

**Authors:** Linhao Wang, Fang Wang, Liling Zhao, Wenjun Yang, Xinxing Wan, Chun Yue, Zhaohui Mo

**Affiliations:** Department of Endocrinology and Metabolism, Third Xiangya Hospital of Central South University, China

## Abstract

**Objective:**

To investigate the effects of mesenchymal stem cells (MSCs) coated by the extracellular matrix (ECM) on wound healing in diabetic rats.

**Methods:**

Mesenchymal stem cells were cocultured with ECM. Cell viabilities were evaluated using MTT assay. The diabetes model was established using both STZ and high-glucose/fat methods in SD rats. A wound area was made on the middle of the rats' back. MSCs or ECM-MSCs were used to treat the rats. HE staining and CD31 immunohistochemistry were used to detect the skin thickness and angiogenesis. Western blotting and qRT-PCR were conducted to determine the level of VEGF-*α*, PDGF, and EGF.

**Results:**

It was observed that treatment of ECM had no significant effects on the cell viability of ECM-MSCs. Wound area assay showed that both MSCs and ECM-MSCs could enhance the wound healing of diabetic rats and ECM-MSCs could further promote the effects. Both MSCs and ECM-MSCs could enhance angiogenesis and epithelialization of the wounds, as well as the expression of VEGF-*α*, PDGF, and EGF in wound tissues, while ECM-MSC treatment showed more obvious effects.

**Conclusion:**

Mesenchymal stem cells coated by the extracellular matrix could promote wound healing in diabetic rats. Our study may offer a novel therapeutic method for impaired diabetic wound healing.

## 1. Introduction

Diabetes mellitus is one of the most prevalent chronic metabolic diseases worldwide. It has been estimated that in 2013, diabetes mellitus has affected over 382 million people worldwide and the incidence is still rising [1, 2]. Diabetes mellitus can cause the impaired wound healing, such as diabetic foot ulcers (DFUs), one of the most common chronic complications, which is also a clinical challenge worldwide [3, 4].

In diabetic patients, the wound healing is often impaired, and the pathophysiology of impaired wound healing in diabetes is complex [5]. Generally, due to the long-term high-glucose condition for tissues and cells in diabetic patients, the skin of diabetic patients is under a higher risk to get damaged and the injury often develops to a chronic, impaired disease which is also prone for recurrence [6, 7]. The long-term high-glucose condition may induce dysfunction of vascular endothelium, dysfunction of macrophages, imbalance of inflammatory reaction, deficiency of growth factors such as vascular endothelial growth factor (VEGF) [8], platelet-derived growth factor (PDGF) [9], and epidermal growth factor (EGF) [10], damage of gelatin, imbalance of epidermal barrier function, and reduction of granulation tissue, which may make the ulcer hard to repair [11, 12].

Mesenchymal stem cells (MSCs), a kind of self-renewing multipotent stem cells, are derived from the bone marrow stroma and other tissues [13]. MSCs are proven to play important roles in many kinds of bioprocesses such as immune, inflammation, and osteogenesis processes [14] and are also involved in gene therapy [15]. Some studies also showed that MSCs had the potential in treatment of diabetes-induced impaired wound healing. It was shown that MSCs could promote the wound healing which was delayed in diabetic mice through promoting epithelialization, as well as increasing angiogenesis and granulation tissue formation [16]. The mechanism may be that MSCs can modify the microenvironment of diabetic patients and thus can be used in treatment of diabetes-induced ulcer [17].

However, studies showed that the therapeutic effect for only MSCs on impaired diabetic wound might gradually reduce with the time, mainly due to the decrease of cell viability during cell migration, leading to worse efficacy for long-term treatment [18, 19]. During the normal wound healing process, the dynamic interaction between damaged cells and its extracellular matrix (ECM) is a crucial part to restore the mechanical integrity of the skin [20]. Studies also showed that restructuring of the extracellular matrix in diabetic wounds might benefit to the wound healing [21]. However, to our best knowledge, up to now no study focused on effects of mesenchymal stem cells coated by the extracellular matrix on wound healing induced by diabetes.

In the present study, we first reported that mesenchymal stem cells coated by the extracellular matrix could promote wound healing in diabetic rats. This study might give deeper understanding for mechanisms of diabetes-induced impaired wound healing as well as provide new potential treatment methods for impaired wound healing.

## 2. Methods and Materials

### 2.1. Cell Culture and Treatment

Human umbilical cord-derived mesenchymal stem cells were isolated and harvested, with ethics committee approval of the Third Xiangya Hospital of Central South University. Cells were cultured in low-glucose Dulbecco's modified Eagle's medium (DMEM) which contained 5 mmol/L glucose, supplemented with 10% Gibco® fetal bovine serum (FBS), 2.0 mmol/L glutamine (Sigma, USA), and 100 *μ*g/mL penicillin-streptomycin (Sigma-Aldrich Co., USA) at 37°C and 5% CO_2_. All cells were divided into 4 groups: the control group (untreated cells), the hypertonic group (cultured with 25 mmol/L mannitol), the high-glucose group (cultured with 30 mmol/L glucose), and the high-glucose and ECM group (cells were premixed with ECM solution for 30 min), and observed under a microscope to make sure they are distributed well. The components of ECM were rat type І collagen (1000 *μ*g/mL) and laminin (500 *μ*g/mL) (Sigma, USA). Cells were cultured to 70–80% confluence for subsequent experiments. MSCs and MSCs cultured with ECM under normal conditions were also obtained and stored for subsequent experiments.

### 2.2. Measurement of Cell Viability

Cell viabilities were evaluated using MTT assay under different time points after treatment, 1 d, 3 d, 5 d, 7 d, and 9 d. Briefly, cells were seeded at density of 4 × 10^4^ in 96-well plates. After 48 h of culturing, 10 *μ*L MTT solution (5 mg/mL) was added. Cells were then cultured for 4 h followed by removal of MTT solution. The supernatant was subsequently replaced with 180 *μ*L DMSO. The optical density (OD) value was evaluated under 490 nm.

### 2.3. Animals and Treatment

SD male rats were purchased from SJA Laboratory Animal Company (Hunan, China). The rats were kept in a light-controlled room under a 12 h/12 h light/dark cycle and controlled temperature (23–25°C). All animals were housed in microisolator cages with free access to food and water according to the *Guide for the Care and Use of Laboratory Animals*. In particular, any effort was put to avoid unnecessary pain of the animals. The whole study was approved by the Institutional Animal Care Committee at Central South University, China. All rats were 7~8 weeks, weighing 180 g–220 g.

The establishment of the diabetes model was performed using both streptozotocin (STZ) (Sigma, USA) and high-sugar and high-fat feeding as reported elsewhere [22]. Briefly, rats were fed with a high-sugar, high-fat diet containing 20% sugar, 10% fat, 4% milk powder, 2% cholesterol, 0.5% sodium cholate, and 63.5% regular diet and the feeding continued for 4 weeks. Then rats were injected with 35 mg/kg STZ. After 72 h of STZ injection, fasting blood glucose (FBS) was tested. The rats were considered to be diabetic with FBS > 16.7 mmol/L in 3 different days. 18 rats were picked out. After establishment of diabetes, a wound area was made for each animal. Rats were first anaesthetized with isoflurane. The dorsal region of rats was then shaved and cleared with iodine complex solution. Subsequently, full-thickness skin defects with diameter of 1 cm in size were created using a sterile surgical scissor on the back (dorsal region) of rats in all groups.

The 18 diabetic rats were then randomized into 3 groups with 6 rats in each group: the control group, in which rats received only 0.5 mL PBS solution by local subcutaneous injection around the wound edge; the MSC group, in which rats received 5.0 × 10^6^ MSCs (suspended in 0.5 mL PBS)/rat by local subcutaneous injection around the wound edge; and the ECM-MSC group, in which rats received 5.0 × 10^6^ ECM-MSCs (suspended in 0.5 mL ECM solution)/rat by local subcutaneous injection around the wound edge. We also picked another 6 diabetic rats and made the same wound without any treatment, to evaluate the natural course of the wound healing. Wounds were monitored by taking digital photos at days 0, 3, 6, 9, and 12 using the Canon PowerShot G9. Animals were restrained by placing on the grid of the cage and held by the base of the tail. Pictures were analyzed by drawing around the wound margins and measuring the pixel area using the ImageJ software; the scabs were included in the wound area.

Rats were sacrificed 12 days after treatments according to Animal Care and Use Guidelines of Central South University. Briefly, rats were first anaesthetized with isoflurane in a closed chamber and then exposed to carbon dioxide for ten minutes to be euthanized. The wound tissue was harvested by dissecting out the skin tissue around the wound margins including epidermis and dermis layers. The specimen was cut into three pieces: one part was fixed in 10% neutral buffered formalin solution and then paraffin embedded for HE staining and immunohistochemistry and the other two parts were flash frozen with liquid nitrogen until total protein and total RNA extraction was performed.

### 2.4. Immunohistochemistry (IHC)

The level of angiogenesis biomarker CD31 was measured by immunohistochemistry according to the previous report [23]. Briefly, the tissue sections were deparaffinized and rehydrated. Antigen retrieval was then performed followed by blocking of endogenous peroxidase through incubation in 3% hydrogen peroxide for 20 min at 37°C. After being blocked by incubating the tissue sections with 4% goat serum for 45 min, the sections were incubated with monoclonal mouse antirat CD31 antibody (NB100-64796, Novus Biologicals, USA, with 1 : 50 dilution) overnight at 4°C, followed by incubation with horseradish peroxidase- (HRP-) conjugated goat anti mouse IgG (sc-2005; Santa Cruz Biotechnology Inc., USA, 1 : 70 dilution) for 45 min at 37°C. Slides were stained using aminoethylcarbazole (AEC) chromogen substrate (AEC Staining Kit; Sigma-Aldrich) for 10 min, and the graphics were observed using the semiautomated computerized ImageJ software (Rasband; NIH, USA).

### 2.5. Quantitative Reverse Transcription PCR (qRT-PCR)

The wound tissues were preserved in liquid nitrogen for RNA isolation. Total RNA was isolated with the TRIzol reagent (Invitrogen, USA) following the manufacturer's instructions. cDNA was synthesized by reverse transcription of total RNA with the RevertAid RT Reverse Transcription Kit (Thermo Fisher). Then, using cDNA as the template, the gene expression levels were analyzed by quantitative PCR conducted on a real-time PCR system (Bio-Rad, USA). PCR were accomplished using the SYBR Green Real-Time PCR Master Mix Kit (Toyobo). The qPCR conditions were 95°C for 5 s and 60°C for 15 s, followed by 70°C for 15 s, for 40 cycles. The relative gene expression level was calculated by the 2^−ΔΔCt^ formula. Primers were synthesized by Sangon Biotech Co. Ltd. The primers were as follows: PDGF-F: 5′-CGCTCCTTTGATGACCTTC-3′, PDGF-R: 5′-GCACTCGGCGATTACGG-3′; EGF-F: 5′-TCGAGTCAACAAAGGGCCTC-3′, EGF-R: 5′-CCCTTCAGCCTGGTTTGCTA-3′; VEGF-*α*-F: 5′-CACCAAGGCCAGCACATAGG-3′, VEGF-*α*-R: 5′-AGGGAGGCTCCAGGGCATTA-3′; and *β*-actin-F: 5′-GAGGGAAATCGTGCGTGAC-3′, *β*-actin-R: 5′-GGAGCCAGGGCAGTAATC-3′.

### 2.6. Western Blotting

Western blotting was conducted to determine the expression of VEGF, PDGF, and EGF in wound tissues of the rats. Briefly, total protein was extracted from the tissues, followed by loading on 10% SDS-PAGE and transferring to PVDF membranes. The membranes were probed with the specific primary antibodies after being blocked with 5% nonfat milk at room temperature for 1 h. All antibodies were purchased from Abcam (Cambridge, MA, USA) as follows: VEGF-*α* (anti-VEGF-*α* antibody, ab53465, dilution 1/1000), PDGF (anti-PDGF antibody, ab23914, dilution 1/1000), and EGF (anti-EGF antibody, ab9695, dilution 1/1000) at 4°C overnight and subsequently incubated with a secondary goat-anti-rabbit antibody (ab97051, dilution 1/5000) at 37°C for 45 min. The target bands were then scanned using enhanced chemiluminescence (Bio-Rad). *β*-Actin was served as a loading control.

### 2.7. Statistical Analysis

The measurement data was expressed by mean ± SD. Comparisons were conducted using one-way analysis of variance (ANOVA) followed by the Tukey post hoc test. It was considered to be statistically significant when *P* value was less than 0.05. All calculations were made using SPSS 18.0.

## 3. Results

### 3.1. ECM Enhanced the Cell Viability of MSCs

**Figure 1:**
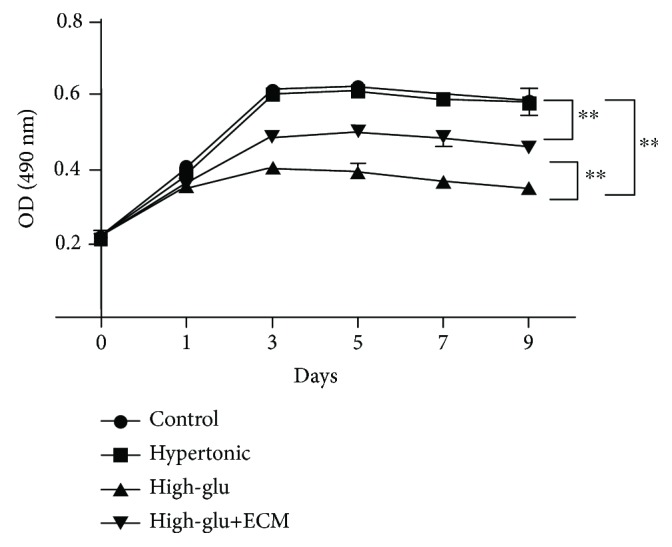
Cell viability in different groups by MTT assay: control—the control group (untreated cells); hypertonic—the hypertonic group (cultured with 25 mmol/L mannitol); high-glu—the high-glucose group (cultured with 30 mmol/L glucose); high-glu+ECM—the high-glucose and ECM group (cells were premixed with ECM). *n* = 3, ^∗∗^ *P* < 0.01.

First, we determined cell viability of MSCs in different groups. As shown in Figure 1, cell viability showed no significant difference between the control group and the hypertonic group. Cell viability in the high-glucose+ECM group was higher than that in the high-glucose group, indicating that treatment of ECM could enhance the cell viability of MSCs. As the ECM solution is a colourless and transparent liquid, the ECM-MSCs just look the same by naked eyes or under a microscope compared with untreated MSCs (Supplementary Figure 1).

### 3.2. ECM-MSCs Could Promote Wound Healing of Diabetic Rats

**Figure 2:**
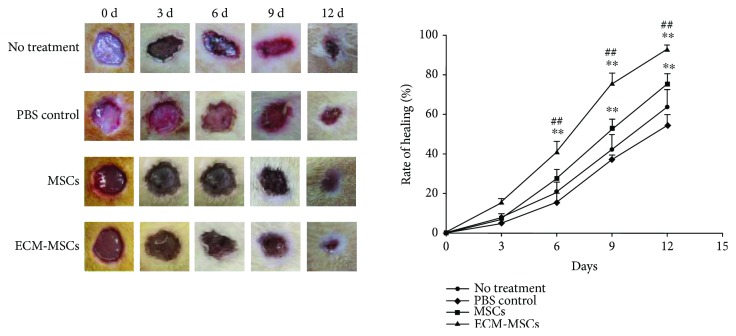
Wound healing at 0, 3, 6, 9, and 12 days for groups of no treatment, control, MSCs, and ECM-MSCs. The rate of healing was the percentage of the reduced wound area vs. the original wound area (%). *n* = 6, ^∗∗^ *P* < 0.01 vs. the control group; ^##^ *P* < 0.01 vs. the MSC group.

As shown in Figure 2, in all groups, the wound areas were reduced gradually. However, when rats were treated with MSCs or ECM-MSCs, the wound areas were obviously smaller since 6 days after treatment compared with those in the PBS control. The wound areas in the ECM-MSC group were apparently smaller than those in the MSC group, indicating that both MSCs and ECM-MSCs might enhance the wound healing of diabetic rats and ECM-MSCs could further promote the effects. Besides, compared with the natural course of the wound healing (no treatment group), the injection of PBS slightly disturbs the wound healing, but there was no significant difference.

### 3.3. ECM-MSCs Could Promote Angiogenesis and Epithelialization of the Wounds of Diabetic Rats

**Figure 3:**
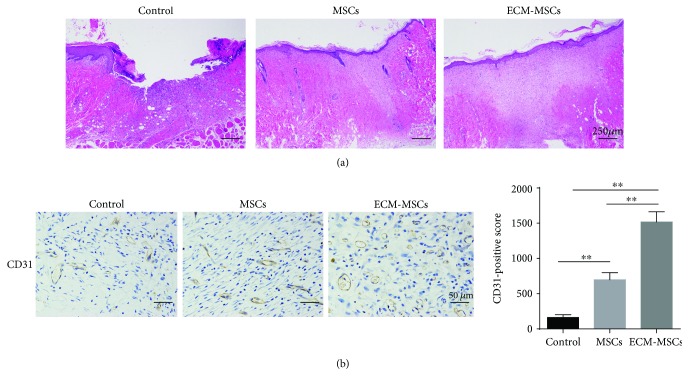
(a) HE staining for wound tissues of different groups of rats (magnification ×40). (b) IHC analysis for the expression of CD31 of wound tissues of different groups of rats (magnification ×200). *n* = 6, ^∗∗^ *P* < 0.01.

To further investigate the effects of ECM-MSCs on wound healing, angiogenesis and epithelialization were tested using IHC and HE staining. Results showed that in both the MSC and the ECM-MSC groups, the tissue damage was obviously reduced and the epithelialization could be apparently observed compared with that in the control group (Figure 3(a)), and epithelialization in the ECM-MSC group was more obvious. Meanwhile, IHC analysis also showed that the expression of CD31 was apparently increased in both MSC and ECM-MSC groups, and the expression was highest in the ECM-MSC group (Figure 3(b)). These results suggested that both MSCs and ECM-MSCs could enhance angiogenesis and epithelialization of the wounds of diabetic rats and ECM-MSCs could further promote the effects.

### 3.4. ECM-MSCs Could Promote the Expression of VEGF-*α*, PDGF, and EGF in Wound Tissues of Diabetic Rats

**Figure 4:**
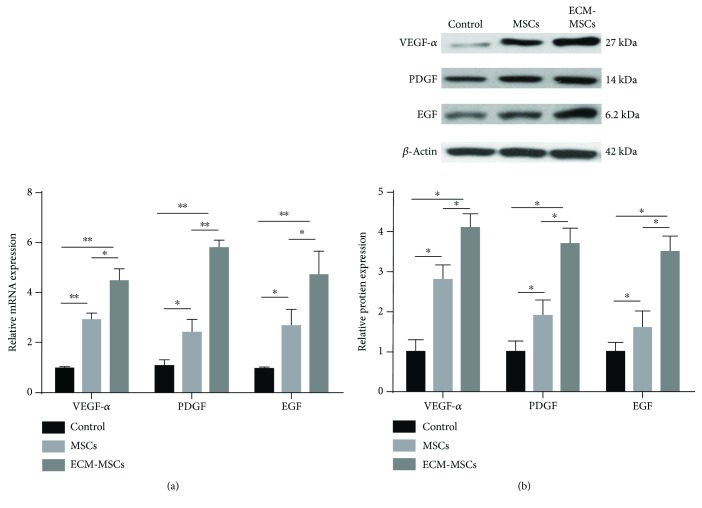
(a) The mRNA level of VEGF, PDGF, and EGF in wound tissues of different groups of rats was quantitated by q-PCR. (b) The protein level of VEGF, PDGF, and EGF in wound tissues of different groups of rats by Western blotting. *n* = 6, ^∗^ *P* < 0.05, ^∗∗^ *P* < 0.01.

Finally, we determined the expression of VEGF-*α*, PDGF, and EGF in wound tissues of diabetic rats. When treated with either MSCs or ECM-MSCs, the protein level and mRNA level of VEGF-*α*, PDGF, and EGF in wound tissues were significantly upregulated compared with those in the control group. Simultaneously, the expression of VEGF-*α*, PDGF, and EGF in the ECM-MSC group was significantly higher than those in the MSC group (Figures 4(a) and 4(b)), indicating that both MSCs and ECM-MSCs could upregulate the expression of VEGF-*α*, PDGF, and EGF in wound tissues of diabetic rats and ECM-MSCs could further promote the effects.

## 4. Discussion

Impaired wound healing is a major diabetic complication which is multifactorial associated with neuronal, vascular, biochemical, and immunological components [20]. It was reported that mesenchymal stem cells could enhance the wound healing, including diabetes-induced impaired wound healing [24]. It was also showed that the extracellular matrix could be used in therapy of chronic wound healings [25, 26]. However, up to now no study focused on effects of mesenchymal stem cells coated by the extracellular matrix on wound healing induced by diabetes.

In the present study, we first reported that mesenchymal stem cells coated by the extracellular matrix could promote wound healing in diabetic rats and enhance the expression of growth factors VEGF, PDGF, and EGF.

First, we investigated the *in vitro* influence of ECM on MSCs and found that treatment of ECM could enhance the cell viability of MSCs under high-glucose culture conditions. Some related studies have been reported. Li et al. demonstrated that mesenchymal stem cells could be influenced by CTGF-VEGF complex in the extracellular matrix [27]. Lozito et al. showed that human mesenchymal stem cells could interact with the endothelial cell matrix in the process for the expression of vascular cell phenotypes [28]. It was also demonstrated that extracellular matrix stiffness could control VEGF signaling and processing in endothelial cells [29].

Then we found that ECM-MSCs could promote wound healing of diabetic rats by promoting angiogenesis and epithelialization. Both ECM and MSCs can enhance the wound healing, and it has been proven in many studies. Choi et al. showed that human placenta-derived extracellular matrix containing bioactive molecules could be used to promote full-thickness skin wound healing [30]. Gao and colleagues demonstrated the promotion effects for diabetic wound healing by a highly bioactive bone extracellular matrix-biomimetic nanofibrous system through rapid angiogenesis [31]. Recently, a clinical study showed treatment potential of early passage autologous mesenchymal stromal cells in accelerating diabetic wound reepithelialization [32]. All these are in consistent with our findings, and we are the first to show that MSCs coated with ECM could promote wound healing of diabetic rats through promoting angiogenesis and epithelialization.

At last, we demonstrated that ECM-MSCs could also promote the expression of VEGF-*α*, PDGF, and EGF in wound tissues of diabetic rats. It is considered that PDGF, VEGF, and EGF have fundamental roles in wound healing [33]. Losi et al. showed that VEGF- and bFGF-loaded nanoparticles could stimulate wound healing in diabetic mice [34]. Choi et al. demonstrated that EGF conjugated with low molecular weight protamine had the potential to treat wound healing [35]. Das et al. found that syndecan-4 could enhance PDGF-BB activity in diabetic wound healing and thus could promote the healing process [36].

How does ECM coating promote these effects? The good results may involve that the ECM components provided the microenvironment for the adhesion and proliferation of MSCs. Some reports indicated that ECM components can improve the viability and biofunction of MSCs. Pei et al. showed that ECM facilitates hBMSC proliferation via selected integrin pathway signals [19]. Shekaran et al. demonstrated that biodegradable ECM-coated microcarriers support human early MSC expansion [37]. Takewaki et al. showed that MSC/ECM cellular complexes induce periodontal tissue regeneration [38]. And a recent study showed that vitamin D and ECM protein FN were able to favor MSC cell adhesion and increase *α*V*β*3 integrin expression [39]. However, the exact mechanism needs further research to explore.

In conclusion, the present study used both *in vitro* and *in vivo* methods to investigate effects of mesenchymal stem cells coated by the extracellular matrix on wound healing in diabetic rats. Results showed that mesenchymal stem cells coated by the extracellular matrix could promote wound healing in diabetic rats through enhancing the expression of growth factors VEGF, PDGF, and EGF. This study might give deeper understanding for mechanisms of diabetes-induced impaired wound healing as well as provide new potential treatment methods for impaired wound healing.

## Data Availability

The data used to support the findings of this study are available from the corresponding author upon request.

## Additional Points

*Highlights*. ECM-coated MSCs promoted wound healing in diabetic rats. ECM-coated MSCs accelerated angiogenesis and epithelialization of the wounds. ECM-coated MSCs increased the expression of growth factors in wound tissues. Our study may offer a promising method for impaired diabetic wound healing.

## Supplementary Materials

Supplementary Materials Figure S1: untreated MSCs and ECM-coated MSCs under an optical microscope: (A) MSCs in PBS (suspended); (B) MSCs coated with ECM (suspended); (C) untreated MSCs in culture; (D) MSCs coated with ECM in culture. Magnification: ×100.

## References

[1] Jeffcoate W. J., Vileikyte L., Boyko E. J., Armstrong D. G., Boulton A. J. M. (2018). Current challenges and opportunities in the prevention and management of diabetic foot ulcers. *Diabetes Care*.

[2] Heublein H., Bader A., Giri S. (2015). Preclinical and clinical evidence for stem cell therapies as treatment for diabetic wounds. *Drug Discovery Today*.

[3] Alavi A., Sibbald R. G., Mayer D. (2015). Diabetic foot ulcers. *Journal of the American Academy of Dermatology*.

[4] Lopes L., Setia O., Aurshina A. (2018). Stem cell therapy for diabetic foot ulcers: a review of preclinical and clinical research. *Stem Cell Research & Therapy*.

[5] Zhang C., Ponugoti B., Tian C. (2015). FOXO1 differentially regulates both normal and diabetic wound healing. *Journal of Cell Biology*.

[6] Bajpai S., Mishra M., Kumar H. (2011). Effect of selenium on connexin expression, angiogenesis, and antioxidant status in diabetic wound healing. *Biological Trace Element Research*.

[7] Ram M., Singh V., Kumawat S., Kant V., Tandan S. K., Kumar D. (2016). Bilirubin modulated cytokines, growth factors and angiogenesis to improve cutaneous wound healing process in diabetic rats. *International Immunopharmacology*.

[8] Zgheib C., Junwang X. U., Hodges M. M., Hu J., Liechty K. W. (2017). Stromal derived factor-1*α* (SDF-1*α*) induces angiogenesis and enhances diabetic wound healing via the miR-15b/VEGF-*α*/BCL2 pathway. *Journal of the American College of Surgeons*.

[9] Choi S. M., Lee K. M., Kim H. J. (2018). Effects of structurally stabilized EGF and bFGF on wound healing in type I and type II diabetic mice. *Acta Biomaterialia*.

[10] Lee C. H., Chao Y. K., Chang S. H. (2016). Nanofibrous rhPDGF-eluting PLGA–collagen hybrid scaffolds enhance healing of diabetic wounds. *RSC Advances*.

[11] Chandu A. (2013). Diabetes, wound healing and complications. *Australian Dental Journal*.

[12] Rafehi H., El-Osta A., Karagiannis T. C. (2011). Genetic and epigenetic events in diabetic wound healing. *International Wound Journal*.

[13] Jiang Y., Jahagirdar B. N., Reinhardt R. L. (2016). Pluripotency of mesenchymal stem cells derived from adult marrow. *Nature*.

[14] Bruder S. P., Jaiswal N., Haynesworth S. E. (1997). Growth kinetics, self-renewal, and the osteogenic potential of purified human mesenchymal stem cells during extensive subcultivation and following cryopreservation. *Journal of Cellular Biochemistry*.

[15] Turgeman G., Pittman D. D., Müller R. (2015). Engineered human mesenchymal stem cells: a novel platform for skeletal cell mediated gene therapy. *Journal of Gene Medicine*.

[16] Jackson W. M., Nesti L. J., Tuan R. S. (2012). Concise review: clinical translation of wound healing therapies based on mesenchymal stem cells. *Stem Cells Translational Medicine*.

[17] Lu H., Wu X., Wang Z. (2016). Erythropoietin-activated mesenchymal stem cells promote healing ulcers by improving microenvironment. *Journal of Surgical Research*.

[18] Hodde J. P., Johnson C. E. (2007). Extracellular matrix as a strategy for treating chronic wounds. *American Journal of Clinical Dermatology*.

[19] Pei M., He F., Kish V. L. (2011). Expansion on extracellular matrix deposited by human bone marrow stromal cells facilitates stem cell proliferation and tissue-specific lineage potential. *Tissue Engineering Part A*.

[20] Ansurudeen I., Sunkari V. G., Grünler J. (2012). Carnosine enhances diabetic wound healing in the db/db mouse model of type 2 diabetes. *Amino Acids*.

[21] Chang M. (2016). Restructuring of the extracellular matrix in diabetic wounds and healing: a perspective. *Pharmacological Research*.

[22] Tian N., Wang J., Wang P., Song X., Yang M., Kong L. (2013). NMR-based metabonomic study of Chinese medicine Gegen Qinlian decoction as an effective treatment for type 2 diabetes in rats. *Metabolomics*.

[23] Gopal A., Kant V., Gopalakrishnan A., Tandan S. K., Kumar D. (2014). Chitosan-based copper nanocomposite accelerates healing in excision wound model in rats. *European Journal of Pharmacology*.

[24] Chen L., Xu Y., Zhao J. (2014). Conditioned medium from hypoxic bone marrow-derived mesenchymal stem cells enhances wound healing in mice. *PLoS One*.

[25] Rando T. (2009). Use of a biological extracellular matrix wound therapy to heal complex, chronic wounds. *Journal of Wound Care*.

[26] Olczyk P., Mencner Ł., Komosinska-Vassev K. (2014). The role of the extracellular matrix components in cutaneous wound healing. *BioMed Research International*.

[27] Li C., Zhen G., Chai Y. (2016). RhoA determines lineage fate of mesenchymal stem cells by modulating CTGF–VEGF complex in extracellular matrix. *Nature Communications*.

[28] Lozito T. P., Kuo C. K., Taboas J. M., Tuan R. S. (2009). Human mesenchymal stem cells express vascular cell phenotypes upon interaction with endothelial cell matrix. *Journal of Cellular Biochemistry*.

[29] Sack K. D., Teran M., Nugent M. A. (2016). Extracellular matrix stiffness controls VEGF signaling and processing in endothelial cells. *Journal of Cellular Physiology*.

[30] Choi J. S., Kim J. D., Yoon H. S., Cho Y. W. (2013). Full-thickness skin wound healing using human placenta-derived extracellular matrix containing bioactive molecules. *Tissue Engineering Part A*.

[31] Gao W., Jin W., Li Y. (2017). A highly bioactive bone extracellular matrix-biomimetic nanofibrous system with rapid angiogenesis promotes diabetic wound healing. *Journal of Materials Chemistry B*.

[32] Maksimova N., Krasheninnikov M., Zhang Y. (2017). Early passage autologous mesenchymal stromal cells accelerate diabetic wound re-epithelialization: a clinical case study. *Cytotherapy*.

[33] Bienert M., Hoss M., Bartneck M. (2017). Growth factor-functionalized silk membranes support wound healing in vitro. *Biomedical Materials*.

[34] Losi P., Briganti E., Errico C. (2013). Fibrin-based scaffold incorporating VEGF- and bFGF-loaded nanoparticles stimulates wound healing in diabetic mice. *Acta Biomaterialia*.

[35] Choi J. K., Jang J. H., Jang W. H. (2012). The effect of epidermal growth factor (EGF) conjugated with low-molecular-weight protamine (LMWP) on wound healing of the skin. *Biomaterials*.

[36] Das S., Majid M., Baker A. B. (2016). Syndecan-4 enhances PDGF-BB activity in diabetic wound healing. *Acta Biomaterialia*.

[37] Shekaran A., Lam A., Sim E. (2016). Biodegradable ECM-coated PCL microcarriers support scalable human early MSC expansion and in vivo bone formation. *Cytotherapy*.

[38] Takewaki M., Kajiya M., Takeda K. (2017). MSC/ECM cellular complexes induce periodontal tissue regeneration. *Journal of Dental Research*.

[39] Posa F., Di Benedetto A., Cavalcanti-Adam E. A. (2018). Vitamin D promotes MSC osteogenic differentiation stimulating cell adhesion and *α*V*β*3 expression. *Stem Cells International*.

