# Erratum to “Mesenchymal Stem Cells Coated by the Extracellular Matrix Promote Wound Healing in Diabetic Rats”

**DOI:** 10.1155/2019/9581478

**Published:** 2019-06-02

**Authors:** Linghao Wang, Fang Wang, Liling Zhao, Wenjun Yang, Xinxing Wan, Chun Yue, Zhaohui Mo

**Affiliations:** Department of Endocrinology and Metabolism, Third Xiangya Hospital of Central South University, China

In the article titled “Mesenchymal Stem Cells Coated by the Extracellular Matrix Promote Wound Healing in Diabetic Rats” [1], due to a production error in Figure 2, the image of day 3 in the MSC group was duplicated to be for day 6 as well. The correct figure should be as follows:

Also, the name of the first author was given incorrectly as Linhao Wang. The author's name should have been written as Linghao Wang. The revised authors' list is shown above.

## Figures and Tables

**Figure 1:**
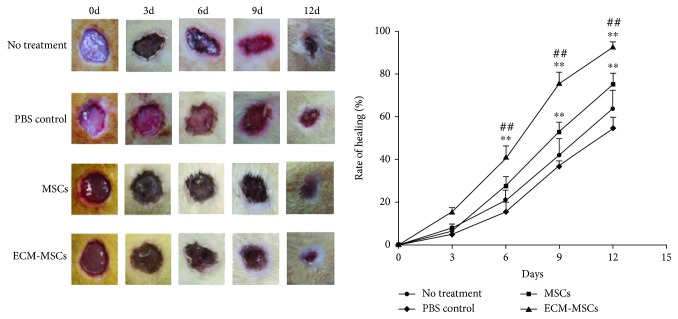
Wound healing at 0, 3, 6, 9, and 12 days for groups of no treatment, control, MSCs, and ECM-MSCs. The rate of healing was the percentage of the reduced wound area vs. the original wound area (%). *n* = 6, ^∗∗^*P* < 0.01 vs. the control group; ^##^*P* < 0.01 vs. the MSC group.

## References

[1] Wang L., Wang F., Zhao L. (2019). Mesenchymal stem cells coated by the extracellular matrix promote wound healing in diabetic rats. *Stem Cells International*.

