# Supplementary material for: Mesenchymal Stem Cells Coated by the Extracellular Matrix Promote Wound Healing in Diabetic Rats
Source: Stem Cells Int. 2019 Jan 28;2019:9564869. doi: 10.1155/2019/9564869 (PMC6369500; doi:10.1155/2019/9564869)
Supplement: Supplementary Materials — Figure S1: untreated MSCs and ECM-coated MSCs under an optical microscope: (A) MSCs in PBS (suspended); (B) MSCs coated with ECM (suspended); (C) untreated MSCs in culture; (D) MSCs coated with ECM in culture. Magnification: ×100. [file 9564869.f1.pdf]

Supplementary Figures

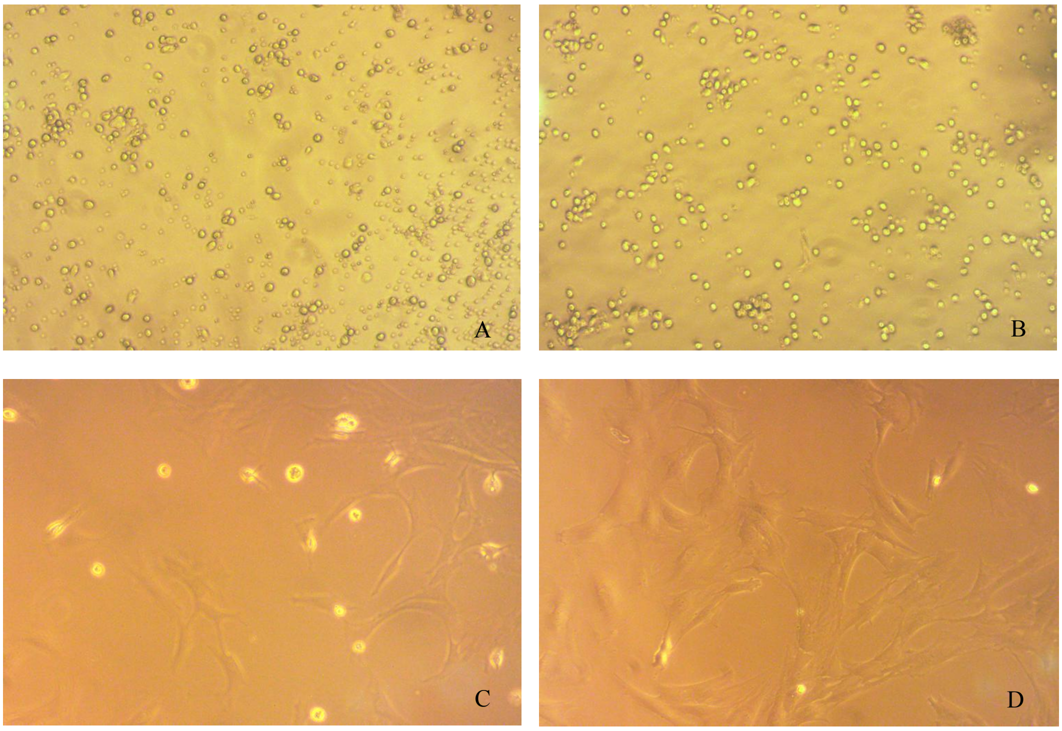

Supplementary Figure 1

Untreated MSCs and ECM coated MSCs under optical microscope. A: MSCs in PBS(suspended); B: MSCs coated with ECM(suspended); C: untreated MSCs in culture; D: MSCs coated with ECM in culture. (magnification: ×100)
